# Detection of Glaucoma in a Cohort of Chinese Subjects with Systemic Hypertension

**DOI:** 10.1155/2013/463710

**Published:** 2013-01-14

**Authors:** Rita A. Gangwani, Jonathan Chan, Jacky Lee, Alfred Kwong, Jimmy S. M. Lai

**Affiliations:** ^1^Department of Ophthalmology, The University of Hong Kong, Hong Kong; ^2^Department of Ophthalmology, Queen Mary Hospital, Hong Kong; ^3^Department of Family Medicine, Hospital Authority, Hong Kong; ^4^COphth Eye Institute, The University of Hong Kong, Room 301, Level 3, Block B, 100 Cyberport Road, Cyberport 4, Hong Kong

## Abstract

*Purpose*. To determine the presence and type of glaucoma in a cohort of adult Chinese subjects with systemic hypertension. *Methods*. This prospective cohort study included 200 hypertensive Chinese adults aged >40 years old who underwent screening via frequency doubling technology (FDT) perimetry and intraocular pressure (IOP) measurement by noncontact tonometry (NCT) in a general outpatient clinic. Those with IOP > 21 mmHg and/or visual field (VF) defects on FDT were referred for complete ophthalmological examination. The diagnosis of glaucoma was based on an abnormal VF on Humphrey Field Analyzer (HFA) by Hodapp-Parrish-Anderson's criteria and an increased vertical cup-disc ratio (VCDR). *Results*. The mean age of the subjects was 64.66 ± 9.47 years, and the male:female ratio was 92 : 108. All patients were hypertensive with a mean blood pressure (BP) of 131.1 ± 15.1/76.6 ± 11.1 mmHg whilst on systemic antihypertensive medication. Of the 111 patients that had an abnormal initial screening, 14 (7.9%) were confirmed to have glaucoma with the highest prevalence of normal tension glaucoma (NTG) (6.2%), followed by primary angle closure glaucoma (PACG) (1.1%) and primary open angle glaucoma (POAG) (0.5%). The positive predictive value of FDT perimetry was 71%. *Conclusion*. Nearly 8% of the adults with systemic hypertension had glaucoma, and NTG was the most prevalent type.

## 1. Introduction

Glaucoma is a progressive optic neuropathy with characteristic changes in the optic nerve head and corresponding visual field loss. Currently, glaucoma accounts for 12% of all global blindness with 4.5 million people affected worldwide [1]. Primary open angle glaucoma (POAG) is more frequent in Caucasian and African populations whilst primary angle closure glaucoma (PACG) is more common in Asian ethnicity. Normal tension glaucoma (NTG) is especially prevalent in Japan and Korea [2–4]. 

Raised intraocular pressure (IOP) remains the most common and clinically modifiable risk factor. However, glaucomatous optic neuropathy can occur despite normal IOP as in NTG [5]. The role of vascular risk factors such as systemic blood pressure (BP) and ocular perfusion pressure, hypercoagulability, carotid artery disease, and vasospasm have been extensively studied, and it has been shown that abnormalities in systemic BP and ocular hypoperfusion are vital in the pathogenesis of NTG [6–10].

Speculations about the association of glaucoma and systemic hypertension still exist. Systemic hypertension has been found to be associated with glaucoma but hypoperfusion has been associated with the development and progression of NTG [8, 11]. The purpose of this study is to determine the presence of glaucoma and type of glaucoma in Chinese adults with systemic hypertension.

## 2. Subjects and Methods

Patients with systemic hypertension and older than 40 years of age attending a regional general outpatient clinic were recruited in the study. Ethics committee approval and informed consent from subjects were obtained for this study.

Hypertension was defined as a presenting systolic BP ≥ 140 mmHg and/or diastolic BP ≥ 90 mmHg without medication. Patients were allowed to rest for 15 minutes before checking BP via an automated digital machine (Dinamap Pro 100, Critikon, Tampa, United States of America). Patients with BP > 160/100 mmHg were rechecked. 

The exclusion criteria consisted of those with central nervous system disease that might affect the perimetry result. 

Patients underwent measurement of IOP with a noncontact tonometry (Topcon Computerized Tonometer CT-80A, Topcon Medical Systems, 111 Bauer Drive, Oakland, NJ 07436, United States of America) and visual field assessment using the FDT perimetry C-20-5 program (Carl Zeiss Meditec, Dublin, CA, United States of America). 

Patients with IOP > 21 mmHg and/or visual field defects on FDT were referred to an Ophthalmology Specialty Clinic where they underwent a complete ophthalmic examination including measurements of best corrected Snellen visual acuity, IOP measurement with Goldman applanation tonometry, gonioscopy, anterior segment examination with slit lamp biomicroscopy, detailed fundus examination including the evaluation of vertical cup disc ratio (VCDR), visual field examination with Humphrey Field Analyzer (HFA) using the 24-2 Swedish Interactive Threshold Algorithm (SITA) (Carl Zeiss Ophthalmic Systems, Inc, California, United States of America), and central corneal thickness measurement with a pachymeter echograph (Quantel Medical, 63039 Clermont-Ferrand cedex 2, France). The presence of glaucomatous optic neuropathy (GON) was established for those with abnormal visual field defects on the HFA according to Hodapp-Parrish-Anderson's criteria together with a corresponding enlarged VCDR [12]. An elevation of IOP was not required for the diagnosis of glaucoma. 

The types of glaucoma were defined as

1. normal tension glaucoma (NTG): GON plus IOP < 21 mmHg and open angle,
2. primary angle closure glaucoma (PACG): GON  plus ≥ 270° peripheral anterior synechiae or appositional closure less than grade 2 on gonioscopy according to the Shaffer grading system,
3. primary open angle glaucoma (POAG): GON  with ≥270° grade 2 or above on gonioscopy according to the Shaffer grading system,
4. Ocular hypertension (OHT): IOP > 21 mmHg without evidence of GON clinically and on HFA.

## 3. Results

Two hundred patients were included in this study. The mean age was 64.66 ± 9.47 years (range 44–88 years). The male-to-female ratio was 92 : 108. All patients were previously diagnosed with hypertension and were started on antihypertensive therapy; they had a mean BP of 131.1 ± 15.1/76.6 ± 11.1 mmHg whilst on antihypertensive medication at the time of the study. 

**Figure 1:**
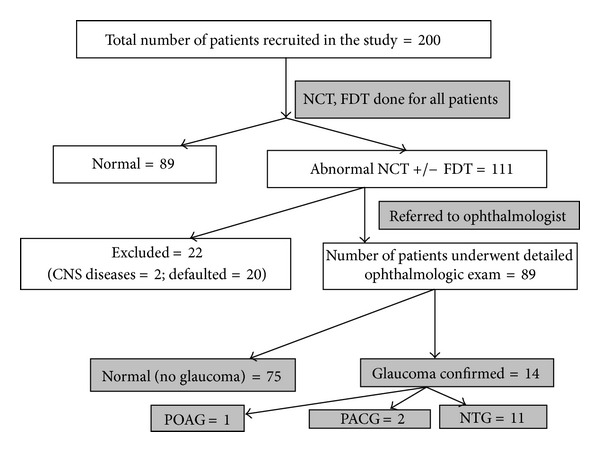
Summary of subjects' inclusion and results.

**Figure 2:**
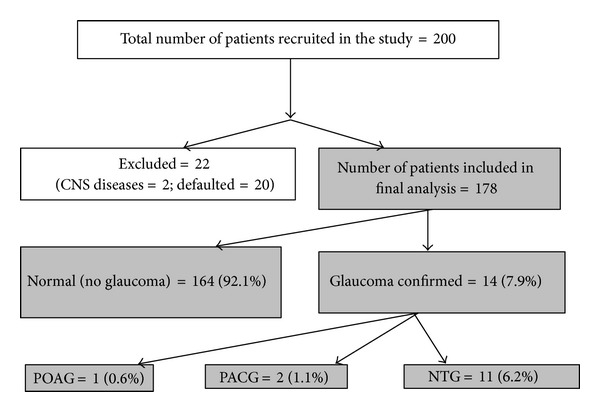
Summary of final results.

Eighty-nine (44.5%) patients had normal IOP and FDT on initial screening. Of the remaining 111 patients that had an abnormal initial screening, 3 (1.5%) patients had an elevated IOP > 21 mmHg, 3 (1.5%) had both an elevated IOP and abnormal FDT perimetry, and 105 patients (52.5%) had an abnormal FDT perimetry only. All 111 patients were referred to an Ophthalmology Specialist Clinic for further assessment. Of these, 2 had coexisting central nervous system disease and therefore were excluded. Twenty patients were lost to followup during the referral process. Of the remaining 89 patients, 14 patients had glaucomatous visual field defects on HFA. Thirty-two subjects had nonspecific visual field defects, and 16 had unreliable visual fields despite repeated investigations. In all, 14 (7.9%) patients were confirmed to have glaucoma. Of these, there was highest prevalence of NTG (6.2%), followed by PACG (1.1%) and POAG (0.5%). OHT (not requiring treatment) was found in 0.5%. Those with nonspecific visual field defects were labeled as glaucoma suspects and on subsequent examinations were diagnosed to be normal (no glaucoma). A summary of participant inclusion and results is shown in Figure 1, while a summary of final results is presented in Figure 2.

The positive predictive value of FDT perimetry was 70% whist the false positive rate was 22.2% with 7.8% of patients unable to produce reliable visual field results. 

## 4. Discussion

There are controversies regarding the role of blood pressure in glaucoma [13–21]. While some studies have indicated that systemic hypertension is not associated with glaucoma [13], others have shown associations between hypertension and POAG and NTG [11, 14]. Even when a correlation exists, some studies correlate systemic hypertension with an increased risk for glaucoma, whilst others report a worsening of glaucoma in those with low blood pressure [11]. Hypertension seems to exert different effects on different types of glaucoma. Hypertension worsens POAG by the elevation of IOP. For every 10 mmHg elevation of systolic and diastolic BP the IOP increases by 0.2–0.44 mmHg and 0.4–0.85 mmHg, respectively [11]. For NTG, however, Wang et al. and Yanagi et al. have shown that blood circulatory disorder plays a more significant role in the pathogenesis [13, 15].

Our study has shown that a substantial proportion (7.9%) of systemic hypertensive Chinese subjects over 40 had glaucoma, with NTG being the most common (6.2%) followed by PACG (1%) and POAG (0.5%). Thirty-two percent of the hypertensive subjects had suspicious HFA results and were followed up as glaucoma suspects for the development of glaucoma. The presence of glaucoma in our systemic hypertensive population in Hong Kong (7.9%) is almost double the prevalence of glaucoma (3.8%) [22] in a general population in Guangzhou, a Chinese province just 170 km away from Hong Kong. Furthermore, our prevalence of PACG is identical (1%) in both populations [22]. On the other hand, the presence of NTG is three times higher than Guangzhou, 6.2% versus 1.8%, possibly signifying an association between systemic hypertension and NTG [22].

The possible relationship between systemic hypertension and NTG can be explained by the postulation that the glaucomatous damage in NTG is caused by unstable perfusion of the optic nerve. Autoregulation is a physiological phenomenon whereby the vascular resistance changes dynamically to keep the blood flow at a constant level to uphold local metabolic demands. Systemic hypertension exerts an oxidative stress to the arterial wall which in addition to atherosclerosis can then impair autoregulation such that an elevation of IOP above a criteria level will comprise the ocular blood flow and induce optic nerve damage even though IOP may still be within normal range [11]. Additionally, when hypertensive patients are started on systemic antihypertensive medication, the diurnal variation, postural hypotension, and nocturnal dips induced by the systemic medication can further comprise ocular perfusion pressure contributing to further optic nerve hypoperfusion [17].

FDT perimetry has been effective for rapid detection of early, moderate and advanced glaucomatous visual field loss [23, 24]. It took approximately 4 to 5.5 minutes to perform an FDT perimetry test using the C 20-5 test pattern. We found that 70% of the subjects with abnormal FDT during the initial screening process had suspicious abnormalities on HFA. Since various factors can contribute to the variability of response during perimetry such as fatigue and learning curve, it is often difficult to diagnose early glaucomatous visual field changes and may take serial perimetries in some cases before definite glaucomatous visual field changes are seen [25–31]. Although HFA is still the gold standard in the diagnosis of glaucoma, our study has shown that FDT perimetry may be used effectively as a simple screening tool in high risk populations and can be easily performed by medical staff without formal ophthalmological training, in a general outpatient setting. 

There were certain limitations of our study. First, there was no local control group for the presence of glaucoma in nonhypertensive subjects. This was because the clinic at which the patients underwent initial screening was a general outpatient clinic which examined the patients with systemic diseases, of which hypertension was one of the most common systemic diseases. Nevertheless we used published data by He et al. from a nearby locality as a basis of comparison to determine the prevalence of glaucoma in the general population [22]. Secondly, 20 out of 111 subjects who were abnormal at initial screening were lost to followup during the referral to the Ophthalmology Specialty centre as subjects were required to pay for the additional investigations in order to be fair to other patients who also require such investigations but not belonging to the study. In addition, although FDT does not have 100% sensitivity to detect glaucoma in a population-based setting, it is likely that some patients with glaucoma may have missed. But we understand that since abnormal FDT and NCT were considered in referring the patients for detailed investigation for presence or absence of glaucoma, it is likely that majority of the patients with glaucoma or suspected glaucoma were included in the detailed examination. An additional fact that needs a mention here is that the subjects with IOP > 21 mmHg were referred (even if they did not have VF defects). So it is likely that we may have missed some patients with lower IOP given the fact that we had a relatively higher prevalence of normal tension glaucoma in our study. But in order to explain this it would be important to consider that IOP of 10–21 mmHg is considered the normal range. Therefore during the screening procedure we considered IOP of >21 mmHg as cut-off point to refer the subjects for detailed ophthalmological examination to determine the presence/absence of glaucoma. This can be considered as a bias in the referral of the patients. But given the fewer number of patients with only IOP > 21 mmHg as the abnormal initial finding, this would not have made significant difference. In spite of these limitations, this preliminary study has served its purpose in highlighting the presence of glaucoma in a systemic hypertensive population. In order to affirm this correlation, a larger scale study involving both nonhypertensive and hypertensive subjects is warranted.

In conclusion, nearly 8% of the systemic hypertensive Chinese adults over 40 were found to have glaucoma with NTG being the most prevalent, followed by PACG and POAG.
